# The relationship of outpatient prescription narcotic use to the early implementation and manner of assisted ventilation in a community hospital intensive care unit

**DOI:** 10.1186/2197-425X-3-S1-A311

**Published:** 2015-10-01

**Authors:** CJ Van Hook, S Burneikiene, D Tangel, B Warner

**Affiliations:** Longmont United Hospital, Intensive Care Medicine, Longmont, United States; Boulder Neurosurgery Associates, Clinical Research, Boulder, United States; Longmont United Hospital, Hospital Medicine, Longmont, United States

## Introduction

The use of prescription opioids has increased in the United States during the past twenty years. The relationship of outpatient opioid use to early assisted ventilation needs in patients admitted to a community hospital intensive care unit was examined.

## Objectives

The purpose of this study was to examine the relationship of outpatient prescription opioid use to the initial ventilatory requirements in patients admitted to a community hospital intensive care unit.

## Methods

Longmont United Hospital (LUH) is a 146 bed institution in Boulder County, Colorado. The intensive care unit is a sixteen-bed combined medical-surgical facility that provides care to any critically ill patient at LUH. Admissions were prospectively studied from January through March of 2015. Patients were evaluated for age, sex, prescription opioid use, and for the type of assisted ventilation required during the initial twenty- four hours of their stay. For purposes of this study, six prescription analgesics: oxycodone, hydrocodone, morphine, fentanyl, tramadol, and hydromorphone were included. Ventilatory assistance was defined as either intubation with mechanical ventilation or the new use of mask delivered bi-level positive airway pressure (BiPAP).

## Results

225 patients were enrolled. The prevalence of outpatient prescription narcotic use was 25.3%. Average ages were 62.9 years in the narcotic group, and 57.5 years in non-narcotic group. The narcotic group demonstrated a sex distribution of 29.8% male, 70.2% female, while the non-narcotic group demonstrated a distribution of 57.7% male, 42.3% female. In the narcotic group, the day-one need for ventilatory assistance was 40.4%, versus 19.1% in the non-narcotic group (p < 0.05) [1]. Among those patients who required ventilatory assistance; 78.3% of patients in the narcotic group received mask BiPAP as the mode of ventilation during the first twenty-four hours of their ICU stay; while in the non-narcotic group, BiPAP was used in 15.6% of those who required assisted ventilation during their first twenty-four hours in the ICU (p < 0.05).

## Conclusions

There is a substantial prevalence of prescribed narcotic use among patients admitted to a community hospital ICU. Patients with pre-existing narcotic use demonstrated an increased need for assisted ventilation during the first twenty-four hours of their ICU stay. There was a significant increase in the use of mask BiPAP as compared to intubation and mechanical ventilation in those patients using outpatient prescription narcotics. Further study is warranted to evaluate ICU length of stay, morbidity, mortality, and cost, as related to the use of outpatient prescription narcotics.
